# Memory, Imagination, and Predicting the Future: A Common Brain Mechanism?

**DOI:** 10.1177/1073858413495091

**Published:** 2014-06

**Authors:** Sinéad L. Mullally, Eleanor A. Maguire

**Affiliations:** 1 Wellcome Trust Centre for Neuroimaging, Institute of Neurology, University College London, London, UK

**Keywords:** imagination, future, episodic memory, hippocampus, scenes, navigation, prospection, scene construction, simulation, prediction, fMRI, neuropsychology, amnesia

## Abstract

On the face of it, memory, imagination, and prediction seem to be distinct cognitive functions. However, metacognitive, cognitive, neuropsychological, and neuroimaging evidence is emerging that they are not, suggesting intimate links in their underlying processes. Here, we explore these empirical findings and the evolving theoretical frameworks that seek to explain how a common neural system supports our recollection of times past, imagination, and our attempts to predict the future.

We predominantly stand in the present facing the future rather than looking back to the past

—Suddendorf and Corballis (2007)

Although not immediately intuitive, the idea that memory, imagination, and predicting what might happen in the future are intimately linked is not new. Throughout the centuries this notion has consistently reemerged within philosophical, psychological, and contemporary work, along with the belief that the role of recollection is to serve imagination and prediction of the future. For instance, in 1798, Immanuel Kant noted that “Recalling the past (remembering) occurs only with the intention of making it possible to foresee the future” (p. 77); in 1871 the White Queen in Lewis Carroll’s *Through the Looking Glass* astutely observed, “It’s a poor sort of memory that only works backwards” (Carroll 1871, chap. 5); whereas in 2006, Suddendorf argued, “It is accurate prediction of the future, more so than accurate memory of the past per se, that conveys adaptive advantage” (p. 1007).

There is behavioral evidence supporting the connection between memory and imagination of the future. For instance, D’Argembeau and Van der Linden (2004) asked participants to mentally “re-experience” personal past events (episodic memory) or to “pre-experience” (episodic future thinking; Atance and O’Neill 2001) possible future events that had/would occur in the close or distant past/future. For the past and future, temporally close events were associated with more sensorial and contextual details and evoked stronger feelings of re-experiencing (or pre-experiencing) than the temporally distant equivalents. Similarly, D’Argembeau and Van der Linden (2006) showed that individual differences, such as capacity for visual imagery, affect the phenomenological experience of episodic memory and episodic future thinking. Notably, specific errors made when recollecting the past are also evident when people engage in predicting the future (for a review, see Gilbert and Wilson 2007).

If memory and imagination are intimately linked, it is natural to ask whether they are supported by the same neural structures. This has been examined in two ways, one with a focus on the hippocampus (Fig. 1) and the other with an eye to an extended set of brain areas—including medial and lateral prefrontal, posterior cingulate and retrosplenial cortices, lateral temporal cortex, and the medial temporal lobes (MTL)—often called the “core network” for episodic memory and imagination (Fig. 2; Buckner and Carroll 2007; Spreng and others 2009). Considering first the hippocampus, since the seminal work of Scoville and Milner (1957), the MTL and in particular the hippocampus have been recognized as playing a pivotal role in our ability to recollect past experiences. Their article described the case of HM who underwent bilateral temporal lobectomy for the relief of intractable epilepsy, rendering him amnesic, unable to acquire new episodic memories. They noted “after the operation this young man could no longer recognize the hospital staff nor find his way to the bathroom, and he seemed to recall nothing of the day-to-day events of his hospital life” (Scoville and Milner 1957, p. 14). The case of HM precipitated 50 years of subsequent work examining the role of the hippocampus in memory (for reviews, see Corkin 2002; Squire 2004).

**Figure 1.**
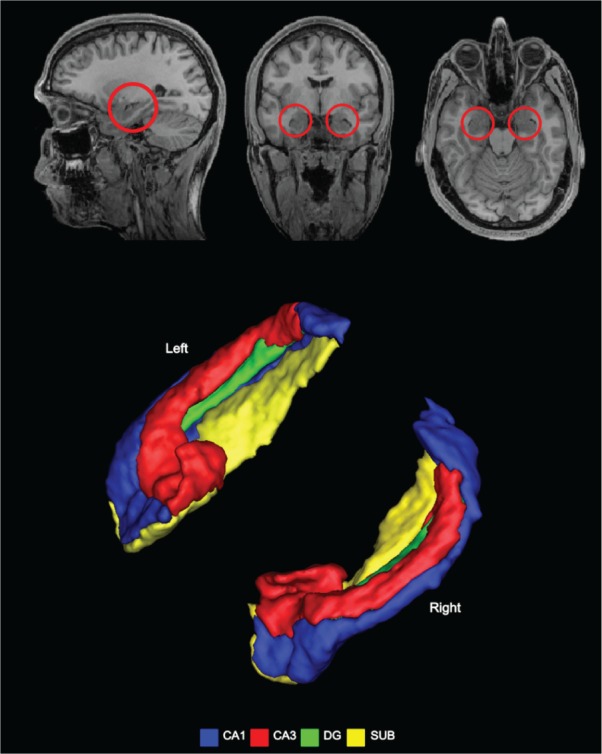
The human hippocampus. The top panel shows the hippocampi circled in red on sagittal (left), coronal (middle) and axial (right) views from a structural MRI brain scan. The hippocampus is composed of a number of subfields, CA1, CA2, CA3, which are adjoined by neighboring areas—the dentate gyrus (DG), the subiculum (SUB), presubiculum, parasubiculum, and entorhinal cortex—to form the extended hippocampal formation. Three-dimensional images of two example hippocampi are shown with some of the subregions indicated (from Bonnici and others 2012).

**Figure 2.**
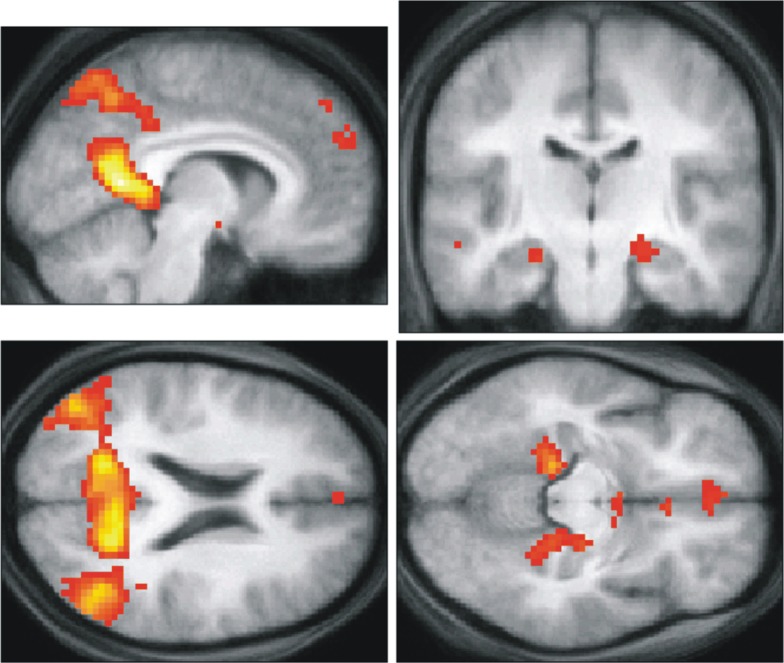
The “core network” for memory and imagination. These are the activations in common for recalling personal past events, recalling previously imagined scenes, and constructing novel scenes (from Hassabis and others 2007a). They are depicted on the averaged structural MRI brain scan of the study’s participants. Areas engaged, relative to baseline control tasks, included lateral and medial prefrontal cortices, precuneus, posterior cingulate, and retrosplenial cortices, lateral and medial temporal areas, including parahippocampal cortex and the hippocampus.

Memory, however, is not the only function that has been ascribed to the hippocampus. In the 1970s, O’Keefe and Dostrovsky (1971) discovered cells in the rat hippocampus that displayed location-specific firing (so-called “place cells”; Fig. 3A), and damage to the hippocampus was found to severely disrupt spatial navigation ability (Morris and others 1982). This evidence prompted O’Keefe and Nadel (1978) to suggest the hippocampus plays a key role in both memory and spatial navigation. Although this idea has been debated (Cohen and Eichenbaum 1991), the onus remains on theoretical accounts of hippocampal function to explain the mnemonic (Spiers and others 2001) and navigation (Fig. 3B; Maguire and others 2006) deficits observed in patients following bilateral hippocampal damage (Burgess and others 2002). But it seems that even explaining memory and navigation is not sufficient; as the links between memory, imagination, and thinking about the future have crystallized, evidence has started to accrue implicating the hippocampus and the core network in these latter functions also. In fact, there has been an explosion of interest in this domain, with Klein (2013) noting a 10-fold increase in investigative activity in the last five years. So what is the evidence that a common neural system, which includes the hippocampus, underpins memory, imagination, and prediction of the future?

**Figure 3.**
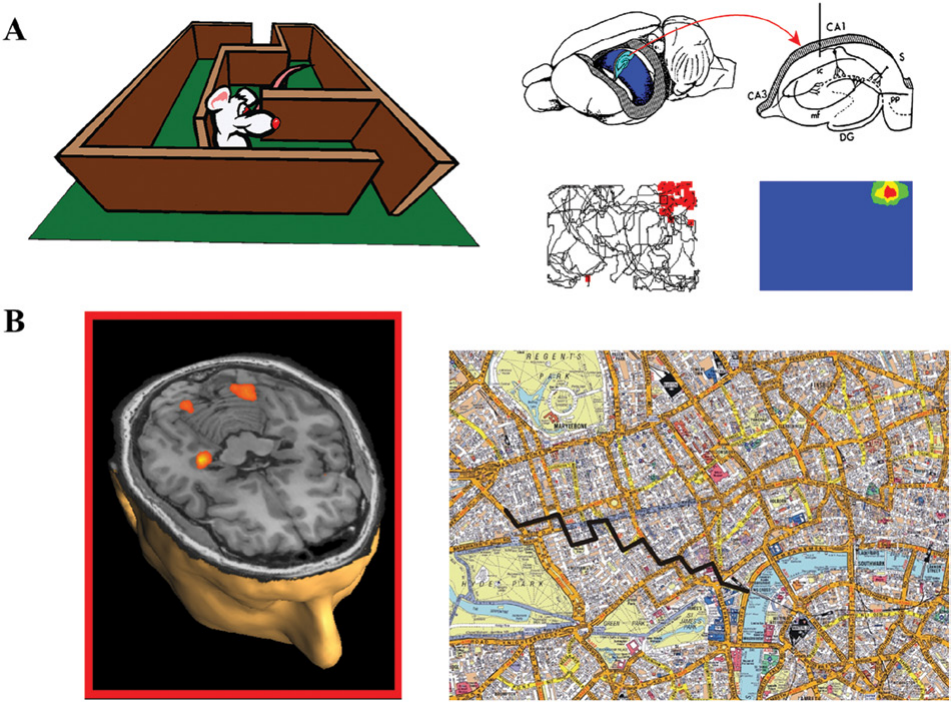
Spatial navigation. (A) Recordings from the hippocampi of freely-moving rats show the presence of place cells that exhibit location-specific firing (Amaral and Witter, 1989, and Burgess and others 1999; reprinted with permission from Elsevier and Oxford University Press). The cell depicted here had its place field in the upper right corner of the arena. (B) When humans navigated routes around a virtual reality version of central London, UK, during fMRI scanning, their hippocampus was engaged (from Spiers and Maguire, 2006). Map reproduced by permission of Geographers’ A-Z Map Co. Ltd. © Crown Copyright 2005. All rights reserved. Licence number 100017302.

## Neuropsychological Evidence

### Patients with Bilateral Hippocampal Damage and Amnesia

Initial interest in the neural substrates of the imagination of future scenarios can be traced to early neuropsychological observations which tentatively suggested that patients with severe amnesia also had difficulties imagining and planning their personal future (e.g., Korsakoff and others 1996; Talland 1965). This paved the way for more detailed investigations. For instance, patient KC who became profoundly amnesic after suffering widespread brain damage (including to the MTL) appeared unable to imagine his personal future (Tulving 1985; see also Rosenbaum and others 2005). Similarly, patient DB displayed episodic memory impairments equal in severity to patient KC’s and was also unable to project himself into the future (Klein and others 2002). As these patients had suffered widespread neurological damage it was not possible to localize the ability to think about the future to specific brain regions.

No formal study was published investigating HM’s ability to imagine fictitious events, but anecdotal evidence suggests his ability to predict his personal future was impaired. When, in 1992, HM was asked what he believed he would do tomorrow he replied “whatever is beneficial” and appeared to have “no database to consult when asked what he would do the next day, week, or in years to come” (S. Corkin, personal communication; cited in de Vito and Della Sala 2011). Similarly, when HM was asked to make a prediction about his personal future, he would respond with a happening from the distant past or he did not respond at all (S. Steinvorth and S. Corkin, personal communication; cited in Buckner 2010). This suggests that the MTL, including the hippocampus, supports the recollection of the past and the imagination of the future.

**Figure 4.**
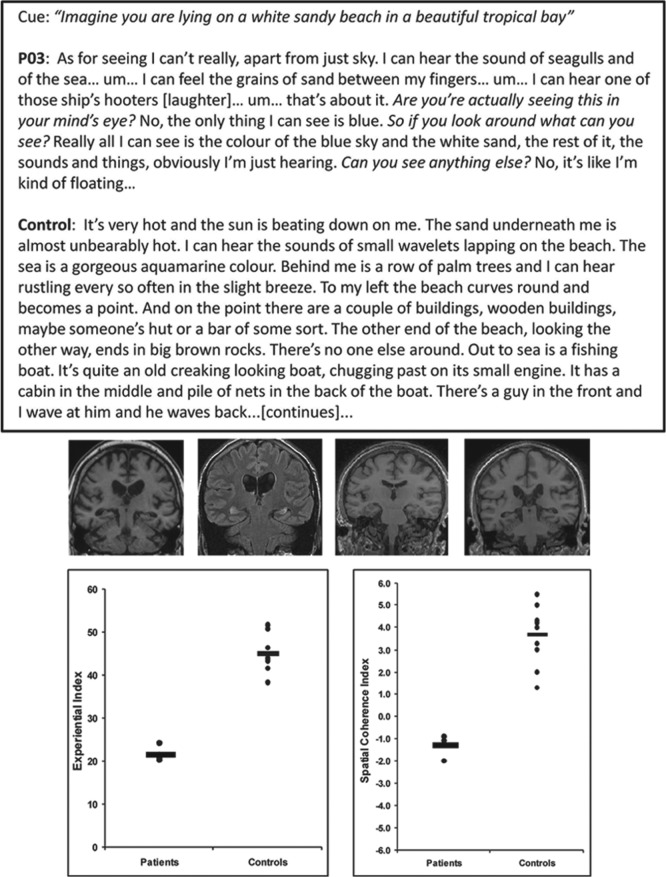
Scene construction in patients with hippocampal damage and amnesia. The top panel shows an example of an imagined scenario from Hassabis and others (2007b). The cue is shown at the top, below which is an excerpt from P03, a patient with bilateral hippocampal damage, followed by that of a control participant who was age-, education-, and IQ-matched to P03. Interviewer’s probing comments are in italics. Relevant background information is noted in square brackets. Underneath are shown coronal sections from the MRI brain scans of four patients, including P03, who were impaired at constructing scenes. The bilateral hippocampal atrophy is notable on each scan. In the lower panel (left) scores on the Experiential Index are provided—this was a measure of the overall richness of the imagined scenes. On the right, scores from the Spatial Coherence Index are given—this was a measure of the spatial contiguity and coherence of the scenes. Data for each of the four patients and every control participant are represented by black dots; horizontal bars indicate the group means.

It was not until 2007, however, that the first systematic study of imagination ability in patients with selective bilateral hippocampal damage was published (Hassabis and others 2007b). These profoundly amnesic patients were unable to construct atemporal fictitious scenes (i.e., scenes with no past or future temporal connotations) in the mind’s eye or to imagine future events involving themselves. For example, when asked to imagine simple fictional scenes such as “imagine lying on a white sandy beach in a beautiful tropical bay” they, unlike controls, struggled to construct a coherent response (Fig. 4). When formally measured, the patients’ scenes were significantly less vivid and more spatially fragmented than those of controls, effects that have since been replicated in a new cohort of seven amnesic patients with focal hippocampal damage (Mullally and others 2012b). Interestingly, this deficit did not appear to be a generalized imagination deficit as the patients were able to vividly imagine single acontextual objects. Nor did it appear to be solely attributable to their memory deficits, because when they were presented with all of the individual scene components required to construct scenes, the patients remained unable to use these elements to form cohesive scene representations (Hassabis and others 2007b). Hassabis and others (2007b) proposed that there was something specific about the imagination of spatially coherent fictitious or future scenes that requires the hippocampus.

Other groups have since reported scene imagination deficits in patients with bilateral hippocampal damage (Andelman and others 2010; Race and others 2011). For example, Race and others (2011) found that amnesic patients with MTL damage were impaired at recollecting their remote and recent past and at imagining their near and distant futures. Critically, these patients were able to generate appropriate and detailed story-based narratives when presented with drawings of scenes, indicating that a generalized narrative problem is unlikely to account for the deficits observed.

Of note, several studies have observed apparently preserved ability to construct scenes in hippocampal-damaged amnesic patients. Mullally and others (2012a) reported the case of P01, who had 50% volume loss along the length of both hippocampi and was densely amnesic, but who was nevertheless able to construct scenes in his imagination. Using fMRI they found that P01’s ability to construct scenes was associated with increased activity in the remnant tissue of his right hippocampus. This suggests that residual function in his lesioned hippocampus was sufficient to support basic scene construction, but this was not enough to rescue his impaired episodic memory which likely requires intact connectivity between the hippocampus and the wider core network. In another study that failed to observe imagination-based deficits in patients with hippocampal damage (Squire and others 2010), the patients were in fact not amnesic, showing instead only mild and non-significant memory deficits when compared with a control group (see more on this in Maguire and Hassabis 2011).

In the preceding patients, hippocampal damage occurred in adulthood. There is another group of patients whose damage is sustained much earlier in life resulting in developmental amnesia. Interestingly, such patients have been found to have a preserved ability to construct scenes (Cooper and others 2011; Hurley and others 2011; Maguire and others 2010). However, it appears that their scene construction is effortful, and may be based on their intact semantic memory and world knowledge (Klein 2013). As such, it does not involve true visualization of an imagined scene, is limited and hippocampal independent.

### Imagination Deficits in Other Populations

Imagination deficits have also been observed in older adults and in other patient groups; populations in which hippocampal and other neural components of the “core network” are known to be compromised (for review, see Schacter and others 2008). In one study, Addis and others (2008) noted deficits in future simulations in older adults. In a subsequent study, Addis and others (2010) sought to investigate whether impairment could simply be an expression of episodic memory deficits whereby elderly adults simply “recast” entire remembered events into the future. Using a recombination paradigm (see also Addis and others 2009) participants first provided a set of autobiographical memories and were later asked to imagine novel events containing a combination of specific details taken from these episodic memories. The older adults continued to generate fewer episodic details for imagined events suggesting that recollection difficulties alone cannot explain their impaired imagination (see also Addis and others 2011; Gaesser and others 2011; Romero and Moscovitch 2012).

Coexisting memory and imagination impairments have also been noted in a number of other populations. Williams and others (1996) reported that suicidal, depressed patients’ recollection of the past and simulation of the future lacked specific details and appeared “over-general” relative to controls. D’Argembeau and others (2008) found that patients with schizophrenia generated significantly fewer episodic details for both past and future events. Interestingly, hippocampal atrophy has been documented in depression (Bremner and others 2000), schizophrenia (Herold and others 2013), and in the aging brain (Driscoll and Sutherland 2005), suggesting that damage to this brain region may be a critical factor underlying these disparate cognitive impairments.

Looking beyond the hippocampus, there have been few neuropsychological studies focused on the role of brain areas outside the MTL in imagination of fictitious/future scenes/events. In one such study, Berryhill and others (2010) tested patients with parietal and prefrontal cortex lesions. Both patient types were impaired, although the precise reasons for this were unclear. The authors suggest, for example, that the frontal patients may have had difficulty with accessing and/or selecting elements for inclusion in the imagined scenes. Nevertheless, this study illustrates that the hippocampus does not act alone in imagination and predicting the future.

## Neuroimaging Evidence

**Figure 5.**
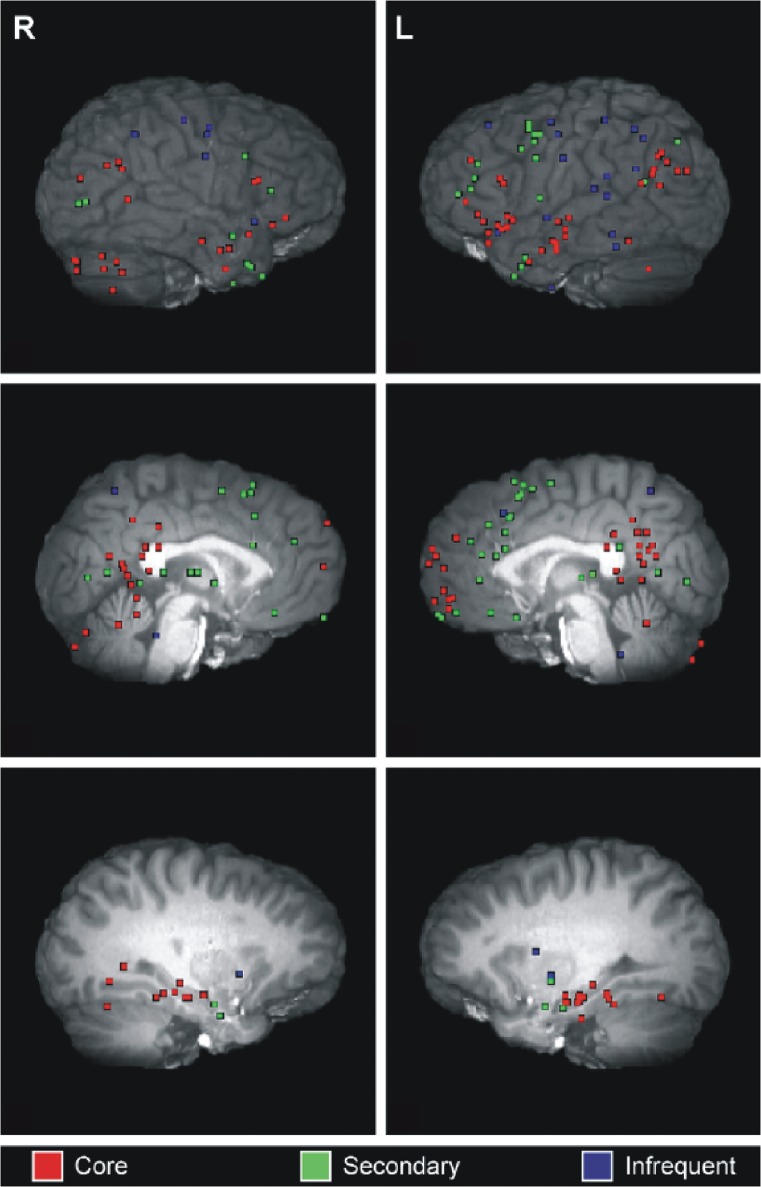
The episodic memory network. Significant peaks of activity from a meta-analysis of 24 neuroimaging studies of autobiographical memory (from Svoboda and others 2006). The classic “core” episodic memory network can be seen in red and includes the hippocampus bilaterally, parahippocampal gyrus, retrosplenial, posterior cingulate and posterior parietal cortices, and medial prefrontal cortex. Activations in core, secondary and infrequently reported regions are depicted across right and left, lateral, medial, and subcortical planes. Reprinted with permission from Elsevier.

A wider network beyond the hippocampus has been highlighted in particular by neuroimaging studies. Episodic memory has been consistently linked with activation of the core network (Fig. 5; reviewed in Maguire 2001; Svoboda and others 2006). Attention then turned to probing whether imagination of fictitious and future events are also supported by a wider set of brain areas beyond the hippocampus, as suggested by the neuropsychological findings, and if these are the same regions that support episodic memory (Okuda and others 2003). Addis and others (2007) showed extensive overlap in activity of this network (the medial and lateral prefrontal, posterior cingulate cortex, retrosplenial cortex, and lateral temporal cortices, and the MTL) when participants recollected or imagined detailed events. This suggests that the episodic system is not solely involved in memory but may also support imagination-based processes. This was later extended to include scene construction processes when Hassabis and others (2007a) reported activation of the core network when participants recollected episodic memories, recollected previously imagined fictitious scenes, or constructed entirely novel fictitious scenes (Fig. 2; see also Addis and others 2009; Botzung and others 2008; Spreng and others; Szpunar and others 2007). In summary, these data, coupled with the neuropsychological evidence, strongly indicate that the core network of brain regions that includes the hippocampus supports both mnemonic and imagination/simulation based processes.

## Theoretical Accounts

### Mental Time Travel into the Future

A number of theories have been proposed which attempt to explain the overlap between memory and imagination. One such account is based on Tulving’s (2002) proposal that recollection of episodic memories involves a mental journey into the past (i.e., mental time travel) in which one has a subjective sense of self over time (“autonoesis”). This concept of mental time travel or self-projection, believed to depend on the integrity of the hippocampus, can also be applied to imagining the future and possibly spatial navigation (Buckner and Carroll 2007; Suddendorf and Corballis 2007; Szpunar and others 2007). This account, however, struggles to explain why hippocampal-damaged patients are unable to imagine atemporal fictitious scenes (Hassabis and others 2007b; Mullally and others 2012b). Indeed, a recent fMRI study found that frontal and parietal cortices, but not the hippocampus, supported mental time travel (Nyberg and others 2010), and Andrews-Hanna and others (2010) showed that imagining scenes best accounted for activity in the hippocampus and MTL, whereas other regions were concerned with the self and with time. Thus, the idea of mental time travel has undoubted heuristic value and may account for the contributions of some areas in the core network to imagining the future, but not the hippocampus.

### Constructive Episodic Simulation Hypothesis

The constructive episodic simulation hypothesis (reviewed in Schacter and others 2012) proposes that episodic memory and thinking about the future are supported by a similar neural network because they are both constructive in nature. In this way, episodic memory is conceptualized as a constructive process (Bartlett 1932), whose critical function is to make available the information required for the simulation/construction of future events. Thus, information is processed in a manner that enables the relevant details to be flexibly recombined to form a novel event. This hypothesized recombination process dovetails with existing theoretical accounts of hippocampal function that emphasize the role of the hippocampus in binding arbitrary or accidentally occurring relations among individual elements within an experience (the relational theory; Cohen and Eichenbaum 1993; Konkel and Cohen 2009) and/or to the specific scene context (the binding of items and contexts model, Ranganath 2010). In this way the constructive episodic simulation hypothesis elegantly explains the co-activation of the core network during self-relevant mnemonic and future simulation processes. However, this account does not explain why patients with hippocampal damage and amnesia have such striking spatial navigation impairments (Burgess and others 2002).

### Scene Construction Theory

**Figure 6.**
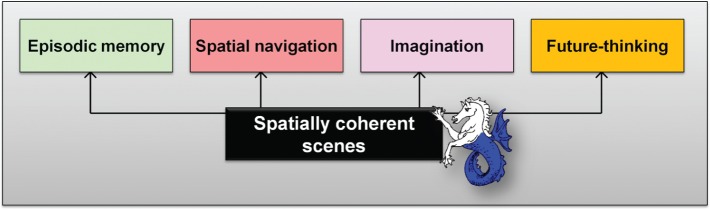
The scene construction theory (Hassabis and Maguire 2007, 2009; Maguire and Mullally 2013) contends that episodic memory, navigation, imagining fictitious scenes and imagining the future (and perhaps even dreaming and mind-wandering) encompass many processes that are not the primary concern of the hippocampus. Nevertheless, it proposes that they each rely on the hippocampus for a critical component which is the construction of spatially coherent scenes.

A third account is the scene construction theory (SCT; Hassabis and Maguire 2007, 2009; Maguire and Mullally 2013), which was originally proposed following the observation that patients with hippocampal damage and amnesia are unable to imagine scenes. In contrast to the constructive episodic simulation hypothesis, which had a broader focus on the core network, SCT attempts to account specifically for the role of the hippocampus in imagination, memory, and spatial navigation. In essence, SCT proposes that the hippocampus primarily acts to facilitate the construction of atemporal scenes and in doing so allows the event details of episodic memories and imagined future experiences a foundation on which to reside. In this way, hippocampal-dependent scene construction processes are held to underpin and support episodic memory, predicting the future, spatial navigation, and perhaps even dreaming and mind-wandering (Fig. 6), with the addition of self-related and temporal processing implemented via the recruitment of other regions within the core network.

Scene construction theory thus places scenes at the center of hippocampal information processing. It does not suggest that the hippocampus is solely responsible for episodic memory, future thinking, and spatial navigation, but rather that the hippocampus supplies a crucial ingredient—scene construction—that they each require (see more on this in Maguire and Mullally 2013). This has intuitive appeal—for most people, recalling the past, thinking about the future, and planning how to get somewhere typically involves imagining scenes. Scenes are also a highly efficient means of packaging information. The SCT also resonates with the patients’ experiences of trying to imagine scenes:

There is no scene in front of me here. It’s frustrating because I feel like there should be. I feel like I’m listening to the radio instead of watching it on the TV. I’m trying to imagine different things happening but there’s no visual scene opening out in front of me.

It’s hard trying to get the space, it keeps getting squashed. (Mullally and others 2012b).

The appeal of the SCT is that it offers a unified account of why such a wide range of seemingly disparate functions are impaired following hippocampal damage.

**Figure 7.**
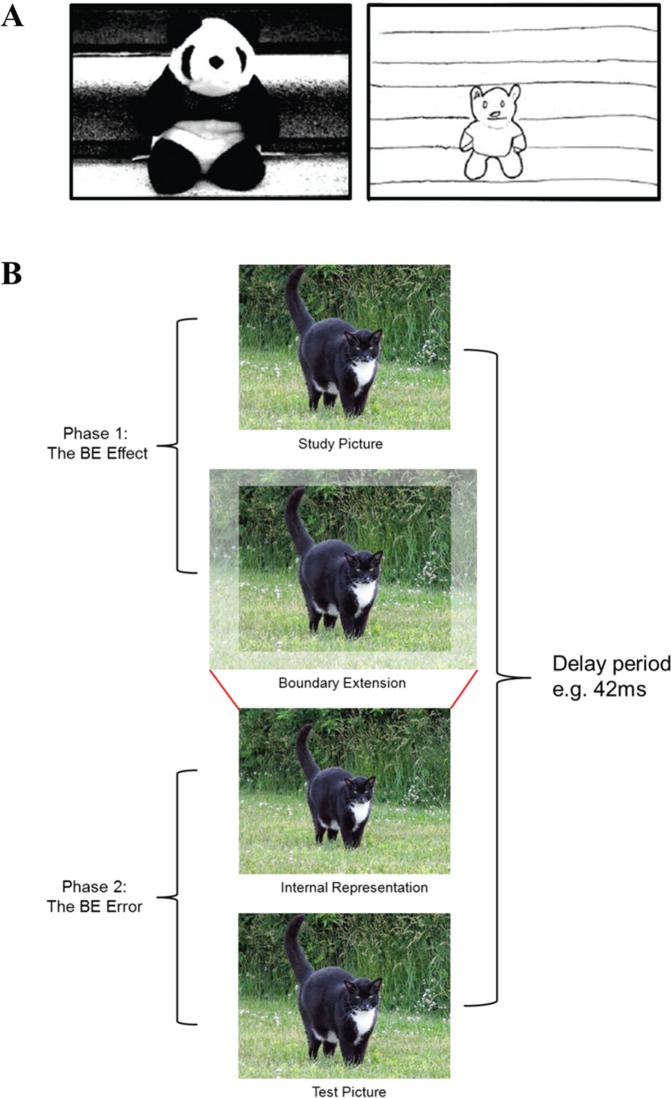
The phenomenon of boundary extension. (A) Healthy participants viewed the picture on the left for 15 seconds. It was then removed and they were required to immediately draw the picture from memory. An example drawing is shown on the right. It is obvious that the participant included much more background than was presented in the picture seconds earlier, thus exhibiting boundary extension (BE; from Intraub and others 1996; reprinted with permission from Elsevier). (B) BE has two phases. When we see a picture of a scene (top panel), we automatically extrapolate beyond the physical edges of that scene (second panel). This active extension of the scene is the “BE effect.” When the scene is no longer present, the extended content and context beyond the boundaries become incorporated into our internal representation of the scene (third panel). Thus, in phase 2, when exactly the same picture is presented at test (fourth panel), we compare the now extended internal representation to the test picture, leading to a perception that the test picture is “closer” than the original study picture, even though they are identical. This memory error is the “BE error,” (from Chadwick and others 2012; reprinted with permission from Elsevier).

Further recent evidence appears to place scene construction at the heart of hippocampal processing. Boundary extension (BE; Intraub and Richardson 1989; Fig. 7A) is a ubiquitous cognitive phenomenon where we erroneously remember seeing more of a scene than was present in the sensory input, and occurs because when we view a scene, we implicitly extrapolate beyond the borders to form an extended representation of that scene. In the absence of the original visual input, this extended scene is misremembered instead of the original input, causing a memory error. BE is a robust and consistent effect found in adults (Intraub and Richardson 1989; Seamon and others 2002), children (Seamon and others 2002) and even babies (Quinn and Intraub 2007). Of note, BE only occurs in relation to scenes and not single isolated objects (Gottesman and Intraub 2002), a dissociation that mirrors the imagination dichotomy observed in amnesic patients (Hassabis and others 2007b).

Boundary extension is composed of two stages (Fig. 7B). The first (the BE effect) requires intact scene construction because it involves the active extrapolation of the scene beyond its physical boundaries resulting in an internally generated “extended scene” representation that persists when the scene is no longer visible. The second phase (the BE error) occurs at retrieval when this internally generated extended scene is conflated with the previously viewed scene from phase 1 producing a memory error. Thus, when people are presented consecutively with exactly the same scene, they consistently judge the scene that is viewed second as being closer-up than the first scene, even though the two scenes are identical. The original scene need only be absent for as little as 42 ms for BE to be apparent, underscoring the online and spontaneous nature of the BE effect (Intraub and Dickinson 2008). Critically, BE depends on an intact ability to construct scenes. Thus, patients with bilateral hippocampal damage and severe amnesia, who are unable to construct scenes should be unable to form this extended representation and therefore fail to commit the BE memory error. This would result in a situation where amnesic patients display superior memory performance relative to healthy controls.

**Figure 8.**
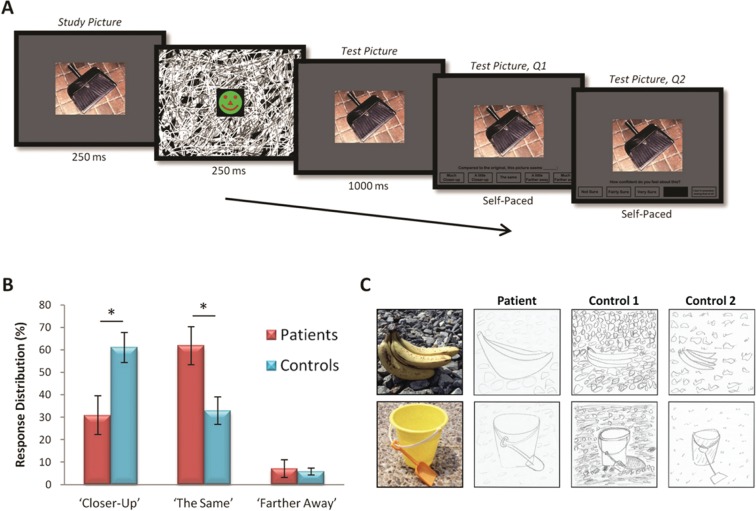
Boundary extension (BE) in patients with hippocampal damage and amnesia. (A) Timeline of an example trial from a rapid serial visual presentation BE task (from Mullally and others 2012b). The initial photograph of a simple scene was presented briefly followed by a dynamically changing mask. The second (test) picture (which unknown to the participants was always identical to the original picture) immediately followed the mask. The task was to rate the second picture relative to the first. There were five options ranging from “much closer-up” to “much farther away,” including the correct response “the same.” (B) BE is revealed by disproportionally larger number of “closer-up” responses. Overall, Mullally and others (2012b) found that control participants made significantly more of these erroneous responses, whereas patients with bilateral hippocampal damage and amnesia made significantly more accurate (i.e., “the same”) responses, and thus showed significantly reduced BE relative to controls. Means (±SEM); *P < 0.05. (C) In a drawing task (also from Mullally and others 2012b) scene photographs (examples shown in the left panel) were studied for 15 seconds and immediately drawn from memory. Drawings by an example hippocampal-damaged amnesic patient (middle left panel) and two matched healthy control participants (middle right and right panels) are displayed. As is evident, this patient more accurately depicted the proportional size of the object relative to the background whereas the control participants’ drawings expose how they extrapolated beyond the given view. Reprinted with permission from Elsevier.

This is exactly what Mullally and others (2012b) recently demonstrated on a variety BE paradigms (Fig. 8). They investigated BE using a rapid serial visual presentation task (Fig. 8A) whereby participants were consecutively presented with two identical scenes and asked to rate the second scene relative to the first (note that on any one trial the two scenes were identical). Seven patients with selective bilateral hippocampal damage and amnesia correctly identified that the study and test pictures were identical with greater frequency than controls, demonstrating more veridical memory (Fig. 8B). They also made significantly fewer BE-driven errors (“closer-up” responses), whereas the number of random errors (“further away” responses) did not differ between the groups.

Participants also performed other BE tasks where they drew simple scenes from memory, and explored and reconstructed scenes while blindfolded using touch alone (haptic task). In both instances the amnesic patients’ BE was greatly attenuated. Overall, therefore, these hippocampal-damaged patients had significantly reduced BE relative to matched controls across a number of independent measures. However, being unencumbered by the BE effect meant that these amnesic patients displayed superior memory to that of non-amnesic controls. Consequently, these results enabled Mullally and others (2012b) to conclude that the patients’ attenuated BE could not be attributed to memory impairment. Therefore, in this context, impaired memory did not lead to impaired scene construction. Instead, impaired scene construction actually lead to better memory; thus successfully separating these two processes.

These data also suggest that a function of the hippocampus is the implicit and continuous prediction of the upcoming environment, that is, the hippocampus is continually constructing scenes, extrapolating beyond the boundaries of our current field of view. A recent neuroimaging study (Chadwick and others 2012) that investigated the role of the hippocampus in BE, supports this hypothesis. Specifically, they found robust activity in the hippocampus during the presentation of the original scene stimulus. Significantly, this activity was observed only on the trials where participants later committed the BE error. This suggests that the hippocampus is involved early at the initial stages of the BE effect where the predictive scene extension processes (attenuated in the hippocampal-damaged patients) are hypothesized to occur. Thus, far from indexing mnemonic impairment, these data (Chadwick and others 2012; Mullally and others 2012b), in addition to the original scene construction findings (Hassabis and others 2007a; Hassabis and others 2007b; Mullally and others 2012b), support the idea that the primary function of the hippocampus may not be mnemonic (Maguire and Mullally 2013; see also Graham and others 2010) but may instead be to predict the nature of the world beyond the immediate sensorium (Bar 2011).

**Figure 9.**
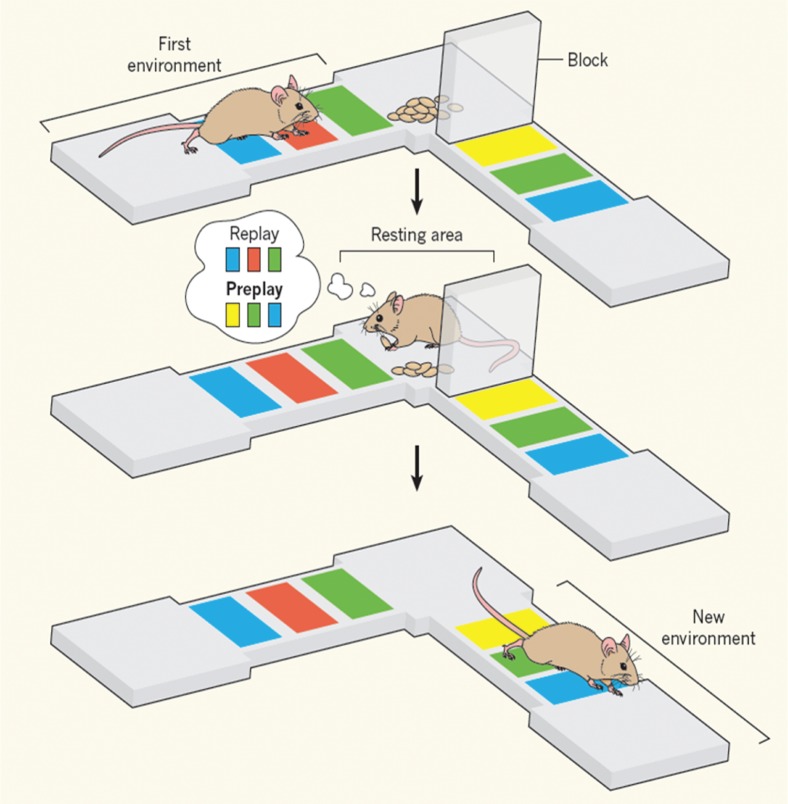
Replay and preplay in the mouse hippocampus. This cartoon (from Moser and Moser 2011, reproduced from *Nature* with permission) shows that when an animal is resting, sequences of neural activity in its hippocampus resemble those that took place during a previous experience, suggesting that the experience is replayed. Dragoi and Tonegawa (2011) have shown that resting mice also preplay activity sequences that are predictive of subsequent activity in environments never visited before.

Interestingly, electrophysiological studies in rodents documenting preplay are starting to add to this evolving picture by hinting at animal parallels in imagining and predicting what might occur in the future (e.g., Diba and Buzsaki 2007; Dragoi and Tonegawa 2011, 2013; Johnson and Redish 2007; Moser and Moser 2011; Fig. 9).

## Conclusions and Future Directions

In conclusion, metacognitive, cognitive, neuropsychological, and neuroimaging evidence clearly illustrate the close ties between episodic memory, imagination and predicting the future. In general, we believe that future studies in humans could greatly benefit from de-conflating two issues—how do we learn and remember our past experiences, and what does the hippocampus do. Despite the hippocampus being widely regarded as the quintessential episodic memory device, as outlined in this article, memory and the hippocampus are not simply interchangeable. By releasing the hippocampus from strictly mnemonic accounts of its function, we believe that a theoretically enriched understanding of its fundamental role and its breakdown in pathology can emerge. To truly ascertain, then, what it is the hippocampus does, a useful strategy going forward, as proposed in the SCT, may be to consider the range of disparate cognitive functions that have been linked to the hippocampus, including memory, imagination, and predicting the future, and deduce from this what common underlying processes or mechanisms may be hippocampally mediated (Maguire and Mullally 2013).

In particular, we need to know more about precisely how the hippocampus supports the construction of scenes, and how this interacts with known computations, such as pattern separation and pattern completion, that occur in its subfields. Does BE occur in non-humans, and if so, what can we learn about the mechanisms involved from electrophysiological studies? Looking beyond the hippocampus, how does hippocampal-dependent scene construction relate to the operation of other cortical areas in the core network such as the parahippocampal and retrosplenial cortices, that are often labeled as “scene-selective” (Auger and others 2012; Mullally and Maguire 2011). What are the precise functions of each area within the core network, and the connectivity between them? There is much yet to learn, and an understanding of the relationship between episodic memory, imagination and predicting the future is still in its infancy. Nevertheless, we are confident that the next five years will hasten important new insights into this question that has intrigued down the ages.
